# Mortality Associated With Viral Bronchiolitis in a Pediatric Department: A Retrospective Analysis

**DOI:** 10.7759/cureus.84571

**Published:** 2025-05-21

**Authors:** Fatima Zahra Alaoui-Inboui, Fatimazahra Yakine, Othmane Ahmito, Nisrine El Merzouki, Soundouss Salimi, Bouchra Slaoui

**Affiliations:** 1 Pneumo-Allergology Unit, Pediatrics Department 2, Abderrahim Harouchi Mother-Child Hospital, Ibn Rochd University Hospital, Casablanca, Casablanca, MAR

**Keywords:** congenital heart disease, death, monoclonal antibodies, prematurity, viral bronchiolitis

## Abstract

Introduction: In Morocco, acute viral bronchiolitis remains a major public health problem, and its incidence continues to rise. Acute viral bronchiolitis can be severe and even fatal, especially in vulnerable populations. The objectives of this study were to analyze the causes of death due to viral bronchiolitis and to highlight the importance of prophylaxis in high-risk groups.

Methods: This was a retrospective, descriptive study spanning 11 years and 11 months, from January 1, 2013, to December 10, 2024. We included all cases of acute bronchiolitis complicated by infant death. The study focused on infants aged one to 24 months who presented with acute bronchitis.

Results: During the study period, 32 cases of viral bronchiolitis resulted in death during hospitalization. The average age of patients was five months and 15 days, with a male predominance. The average duration between the onset of symptoms and death was seven days, ranging from 24 hours to 30 days. The risk factors included male sex (n=20, 62.5%), passive smoking (n=17, 53.1%), young age (n=16, 50%), and preterm infancy (n=4, 12.5%). Comorbidities were found in 25 (78%) cases, including 19 (59.4%) of congenital heart disease, one (3.12%) of bronchopulmonary dysplasia, one (3.12%) of spinal muscular atrophy, one (3.12%) of hypopituitarism, one (3.12%) of ichthyosis, and one (3.12%) of polymalformative syndrome. The primary causes of death were congenital heart diseases, including ventricular septal defect (n=5, 15.62%), dilated cardiomyopathy (n=3, 9.37%), complex congenital heart disease (n=1, 3.12%), double outlet right ventricle (n=1, 3.12%), and tetralogy of Fallot (n=1, 3.12%). The average duration of hospitalization was five days, ranging from one hour to 15 days. All patients required intensive care, but they could not be transferred due to the lack of available beds, leading to mortality.

Conclusion: Infants born prematurely with chronic lung disease or with decompensated congenital heart disease are at increased risk of severe acute bronchiolitis, particularly due to respiratory syncytial virus (RSV). This risk underscores the importance of prophylaxis with anti-RSV monoclonal antibodies.

## Introduction

Acute lower respiratory infections (LRIs) remain a challenge to children’s health worldwide, especially in developing countries where access to healthcare is scarce. In children under five years of age, LRI is the most frequent cause of death, including viral bronchiolitis in young infants. Viral bronchiolitis is an acute viral respiratory infection that affects 30% of infants under two years of age [1], with massive epidemics occurring yearly during the autumn-winter season. Although viral bronchiolitis is generally mild, some cases are severe [2]. We recall that benign viral bronchiolitis is easy to diagnose. It is characterized first and foremost by the parents' observation of an unusual cough in the infant, or even some respiratory discomfort. On clinical examination, the infant's general condition is preserved, dyspnea is predominantly exhalatory, and appetite is undisturbed. It may be associated with polypnoea, wheezing audible at a distance, sibilants, or crackles on auscultation, but without signs of respiratory distress.

The primary cause of viral bronchiolitis is the respiratory syncytial virus (RSV), which accounts for 50% to 80% of acute bronchitis cases [3]. Severe RSV cases are associated with high risks of hospitalization and complications, causing a significant concern for young parents. In addition, the mortality rate associated with this infection remains high, especially in developing countries [4], with an estimated 66,000 to 199,000 deaths occurring annually worldwide [5].

Young infants, especially those with pre-existing cardiorespiratory problems, are at increased risk of acute respiratory failure requiring mechanical ventilation and/or potentially life-threatening conditions. Predisposing factors of death from acute bronchiolitis include congenital heart disease, chronic lung diseases, and prematurity [2].

In this study, we aimed to identify the risk factors for mortality in infants with severe bronchiolitis and to highlight the importance of prophylaxis in high-risk infants.

## Materials and methods

Study design

This retrospective, descriptive study spanned 11 years and 11 months, from January 1, 2013, to December 10, 2024. The study population included infants under 24 months who were hospitalized in the pediatric department of Abderrahim Harouchi Mother-Child Hospital, Casablanca, Morocco, during the study period.

Inclusion and exclusion criteria

All infants aged one to 24 months who presented with acute bronchiolitis were included in the study. Infants with incomplete medical records were excluded. The diagnosis of acute bronchiolitis was based on the first episode of acute dyspnea with rhinorrhea followed by cough, wheezing on auscultation with or without tachypnea, and/or signs of respiratory distress occurring at any time of the year. We included the files of infants who had died of viral bronchiolitis on the ward and not those who were transferred to and died in the intensive care unit.

Data collection

Clinical data were collected from the medical records of patients hospitalized with viral bronchiolitis.

Statistical analysis

Statistical analysis was carried out using Microsoft Excel (Microsoft Corporation, Redmond, Washington, United States), in which the percentage of deaths relative to the total number of viral bronchiolitis cases hospitalized during the same period was determined. For the descriptive analysis, the results are presented in absolute numbers, and percentages are used for qualitative variables.

## Results

During the study period, 6451 infants aged one to 24 months were hospitalized due to acute bronchiolitis. Among them, 32 infants died, resulting in a mortality rate of 0.5%. The patients' average age was five months and 15 days. Viral bronchiolitis affected 13 infants under three months of age and three infants between three and six months of age, with a male predominance (male-to-female ratio of 1.6). Regarding medical history, among the 32 deceased infants, four (12.5%) were preterm infants under 36 weeks of gestation, five (15.6%) were exclusively breastfed, eight (25%) were not properly vaccinated according to the national immunization program, and 17 (53.1%) were exposed to passive smoking (Table 1).

**Table 1:** Risk factors associated with mortality due to viral bronchiolitis.

Risk factor	Number of cases
Young age	16 (50%)
Male sex	20 (62.5%)
Passive smoking	17 (53.1%)
Preterm infant	4 (12.5%)

Clinically, the average time between symptom onset and hospitalization was seven days, ranging from 24 hours to 30 days. The initial clinical presentation included severe respiratory distress in all patients. Among them, seven (21.87%) patients presented with cyanosis, 17 (53.1%) exhibited tachypnea, and 21 (65.6%) displayed signs of respiratory distress. Twenty-one (65.6%) infants were hypoxic with an oxygen saturation below 91% on room air. Two (6.25%) infants were hospitalized with sepsis and hemodynamic instability disorders. Nineteen (59.3%) infants were underweight, and six (18.75%) had a heart murmur detected on cardiovascular auscultation. All infants had feeding difficulties (Table 2).

**Table 2:** Clinical signs of hospitalized patients for viral bronchiolitis.

Clinical sign	Number of cases
Respiratory distress	32 (100%)
Cyanosis	7 (21.87%)
Feeding difficulties	32 (100%)
Tachypnea/apnea	17 (53.1%)
Signs of respiratory distress	21 (65.6%)
Toxic appearance	2 (6.25%)
Fever	2 (6.25%)
Heart murmur	6 (18.75%)
Underweight	19 (59.3%)
Thoracic deformity	1 (3.12%)
SpO2<91%	21 (65.6%)

**Figure 1:**
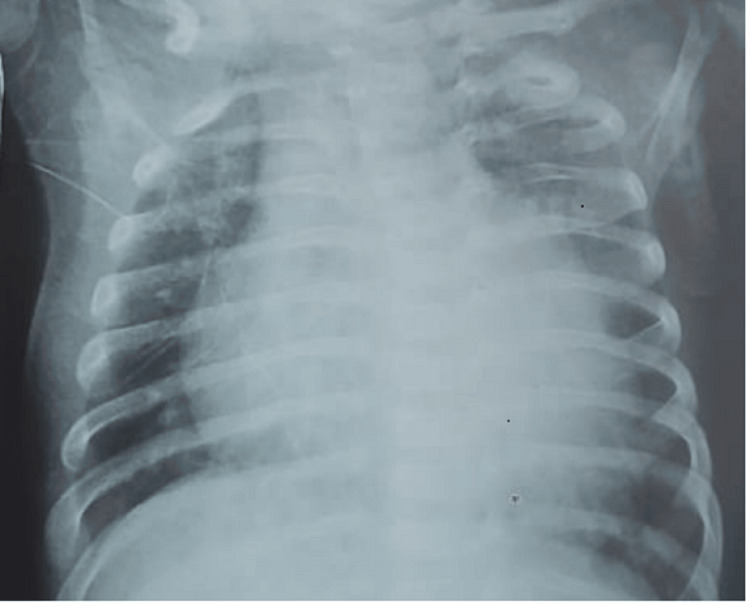
Cardiomegaly with a cardiothoracic ratio of 0.63 in a 10-month-old infant admitted for severe viral bronchiolitis who died on the 10th day of hospitalization due to heart failure.

**Figure 2:**
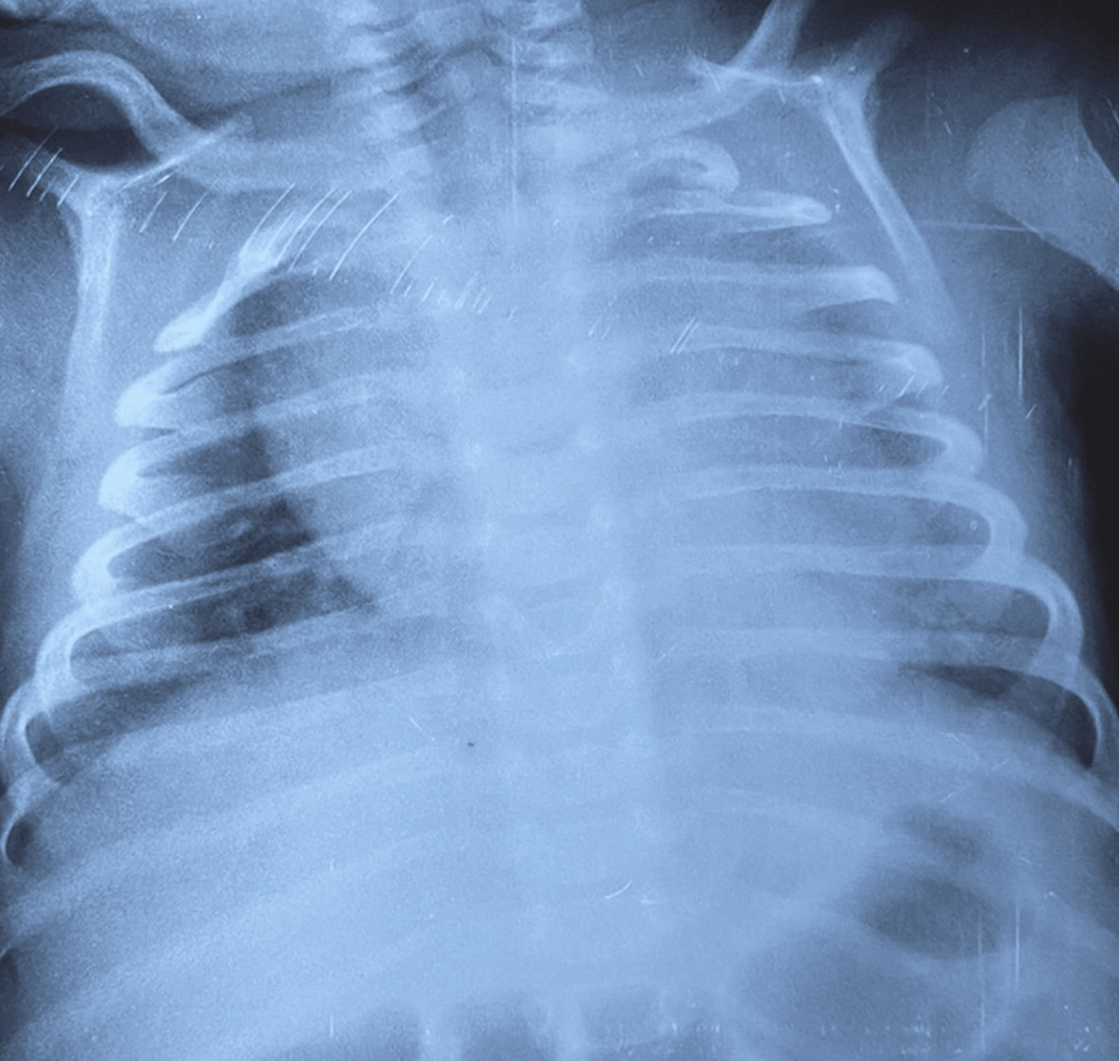
Cardiomegaly with a cardiothoracic ratio of 0.62 in a 40-day-old infant admitted for severe viral bronchiolitis who died on day seven of hospitalization due to sepsis.

**Figure 3:**
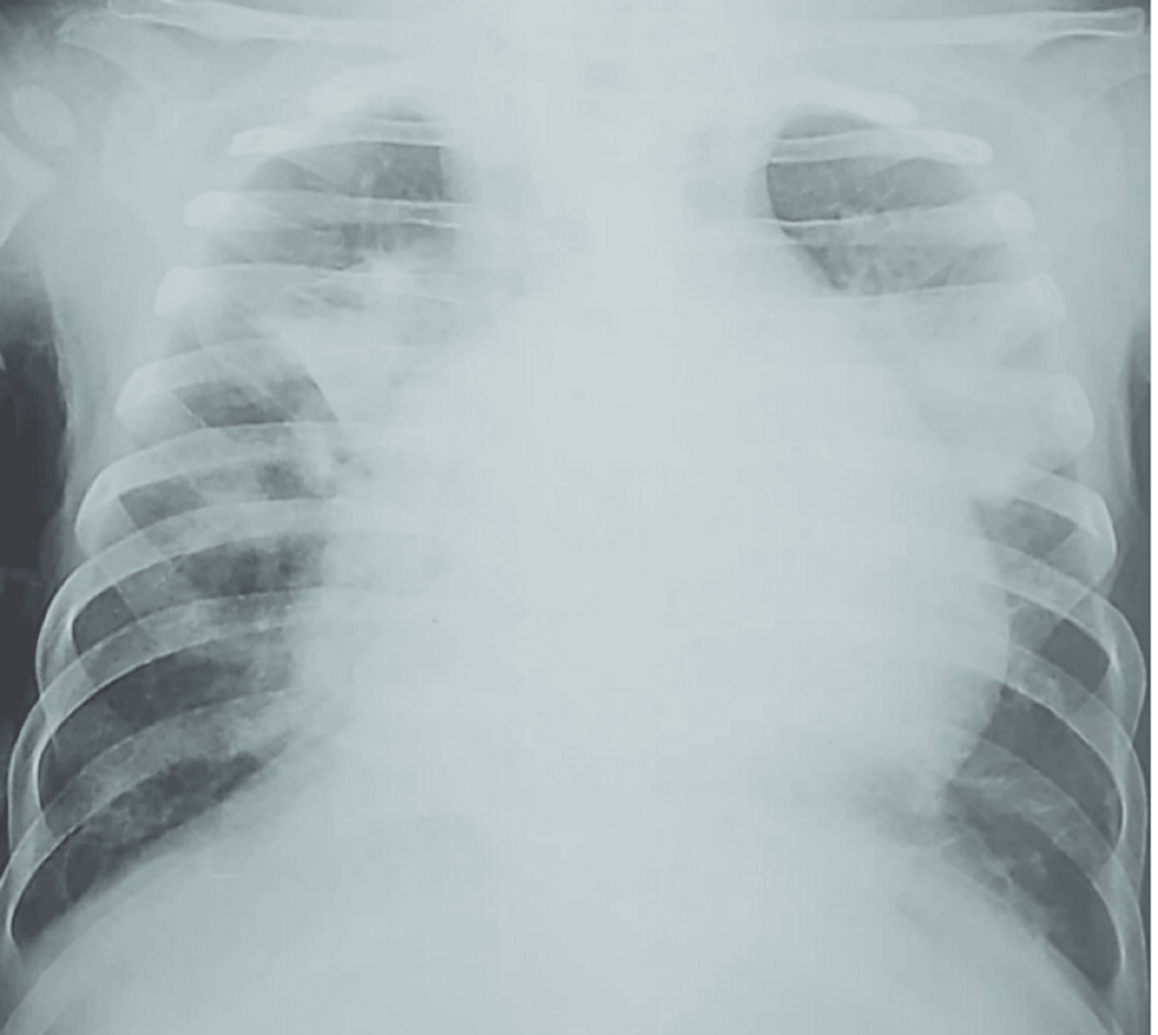
Bilateral alveolar opacity in a 12-month-old infant admitted for severe bronchiolitis who died on day five of hospitalization due to sepsis.

Chest X-rays revealed cardiomegaly in 19 (59.3%) cases (Figures 1, 2), as well as alveolar opacity in 15 (47%) cases (Figure 3). In addition, chest X-rays showed a thoracic deformity due to spinal muscular atrophy in one infant (3.1%)

Echocardiography was performed on 11 (34.37%) infants, which revealed a ventricular septal defect in five (45.3%) patients, three of whom (27.2%) had severe pulmonary hypertension, and two (18.1%) had right ventricular dilation and hypertrophy. Three (27.2%) patients had dilated cardiomyopathy, and one (9%) patient had complex heart disease, with double outlet right ventricle and tetralogy of Fallot (9% each).

Biologically, neutrophilic leukocytosis was documented in 10 (31.25%) patients. Four (12.5%) patients had neutrophil counts between 8000 and 10000/mm, four (12.5%) had counts between 10000 and 15000/mm, and two (6.25%) had counts above 15000/mm. Elevated C-reactive protein was found in 10 (31.25%) patients, whereas this value was between 50 mg/L and 70 mg/L in eight (25%) patients. Twenty-five (78%) infants had comorbidities, including congenital heart disease (n=19, 59.4%), bronchopulmonary dysplasia (n=1, 3.12%), and polymalformative syndrome (n=1, 3.12%) (Table 3).

**Table 3:** Comorbidities of patients hospitalized for viral bronchiolitis.

Comorbidity	Number of cases
Decompensated congenital heart disease	19 (59.4%)
Bronchopulmonary dysplasia	1 (3.12%)
Spinal muscular atrophy	1 (3.12%)
Neonatal lupus	1 (3.12%)
Hypopituitarism	1 (3.12%)
Ichthyosis	1 (3.12%)
Polymalformative syndrome	1 (3.12%)

In this study, the primary cause of death was viral bronchiolitis with congenital heart disease in 19 (59.4%) cases. Death from sepsis due to the secondary bacterial infection of viral bronchiolitis occurred in eight (25%) cases. The other causes of death were viral bronchiolitis with spinal muscular atrophy (n=1, 3.12%), complex brain malformation with hypopituitarism (n=1, 3.12%), ichthyosis (n=1, 3.12%), and polymalformative syndrome with biliary atresia (n=1, 3.12%) (Table 4).

**Table 4:** Causes of mortality in viral bronchiolitis.

Causes of mortality	Number of cases
Bronchiolitis with congenital heart disease	19 (59.4%)
Sepsis due to superinfected bronchiolitis	8 (25%)
Bronchiolitis with spinal muscular atrophy	1 (3.12%)
Bronchiolitis with neonatal lupus	1 (3.12%)
Bronchiolitis with hypopituitarism	1 (3.12%)
Bronchiolitis with ichthyosis	1 (3.12%)
Bronchiolitis with polymalformative syndrome	1 (3.12%)

The average duration of hospitalization was five days, ranging from one hour to 15 days. All patients required intensive care but could not be transferred due to the lack of available beds, resulting in mortality.

## Discussion

Viral bronchitis represents the main autumn-winter epidemic in pediatrics. This infection poses a major challenge for healthcare systems as it constitutes a significant economic burden, particularly in low-income countries, and because it is considered the main risk factor for severe morbidity and mortality in young children, particularly those under the age of two years.

Viral bronchiolitis is mainly caused by RSV, which is found in 50-80% of cases [6]. With an R naught (R0) of approximately 4.5, this virus is highly contagious. It is so ubiquitous, especially in community settings, that almost all infants will be infected by this virus before the age of 24 months [7]. RSV generally affects infants under two years of age, with a peak incidence between two and six months [8]. In France, 3% of infants with viral bronchiolitis require intensive care hospitalization [9], which is consistent with findings in the United Kingdom, where approximately one in three infants develop viral bronchiolitis in the first year of life, of which 2% to 3% are hospitalized [8].

According to a meta-analysis by Bont et al., viral LRIs (e.g., bronchiolitis) accounted for 12% to 62% of hospitalizations in Western countries [10]. Among these cases, 2-12% were admitted to intensive care units, with a mortality rate of <0.5%, which is similar to that found in this study. In another meta-analysis of infants under the age of one year living in developed countries and hospitalized for severe viral bronchiolitis, the mortality rate was 0.7% [5]. In contrast, a 2014 study conducted in Colombia found that RSV-related respiratory infections accounted for 33.8% of hospitalizations, with a mortality rate of 1.1% [1]. In Madagascar, a study lasting over four years involving 1827 infants hospitalized for viral bronchiolitis reported 36 deaths, yielding a mortality rate of 1.9% [2], which is relatively high compared to developed countries. In France, in 2009, 29,784 hospitalizations of infants under the age of one year for acute bronchiolitis led to a mortality rate of 0.08% [11]. In a literature review, the mortality rate of RSV infections was less than 1% among infants without risk factors or comorbidities. The highest rate was observed among children admitted to intensive care and requiring respiratory support [12].

There are very specific criteria that should lead to the hospitalization of infants with acute bronchiolitis. Many studies show that the annual number of infants requiring hospitalization is high, especially during the winter season [11]. In this study, the total number of hospitalized infants was 6451, and we quantified the number of deaths related to bronchiolitis among infants hospitalized for viral bronchiolitis. Male predominance was observed in this study, with a male-to-female ratio of 1.3 [13]. Similarly, the French Institute for Public Health Surveillance noted a male predominance (59% of cases) in those hospitalized for viral bronchiolitis in 2012 [8].

The risk factors identified in infants who died from viral bronchiolitis could be considered indicators of severe forms of bronchiolitis in infants. In a 2005 study conducted in Paris by Chevret et al. [14], prematurity, young age at admission, and the occurrence of acute respiratory distress syndrome were considered the main risk factors for death related to viral bronchiolitis. Other risk factors have been identified, including low birth weight, artificial feeding, and exposure to passive smoking [11]. In their study conducted at St. Mary’s Hospital in London, UK, Ghazaly suggested that RSV infection in young infants is also a risk factor for death from viral bronchiolitis [15]. A Taiwanese study on risk factors associated with death in patients with RSV infection found that nearly 50% of patients admitted to intensive care were preterm, of which 25% had contracted a nosocomial RSV infection [16]. Unfortunately, in this study, we observed similar risk factors, including male predominance (20/32 cases), passive smoking (17/32 cases), young age at admission (16/32 cases), and former preterm infants under 36 weeks of gestation (4/32 cases). However, only five infants were exclusively breastfed.

Several studies have attempted to explain the link between the severity of viral bronchiolitis episodes and the young age of infants at the time of hospital admission. Due to their physical and immunological immaturity, infants under six months of age have the highest exposure to severe viral bronchiolitis. In a cohort of infants under one year of age in Lyon hospitalized during the winter of 2016-2017, young age was a risk factor associated with the severity of viral bronchiolitis [17].

Breast milk is known for its richness in nutrients and protective elements for infant health. A 2013 study done in Italy demonstrated that breastfeeding, even in combination with artificial feeding, reduced the risk of hospitalization for viral bronchiolitis during the first year of life [18]. Another study on the same subject showed that infants who were not breastfed had three times the risk of hospitalization for LRI compared to those exclusively breastfed for four months [19].

In a cohort study based in the UK, Thorburn demonstrated that only infants with comorbidities died from severe RSV infection [20]. In a 2018 study conducted by Ghazaly in the UK, among 274 infants hospitalized for viral bronchiolitis in intensive care, the main associated comorbidities were gastroesophageal reflux (47.17%), cardiac anomalies (44.16%), chronic lung disease including bronchopulmonary dysplasia (13%), and neurological anomalies (7%) [15]. According to Rakotonoel et al., the presence of congenital heart disease in infants with bronchiolitis may increase their risk of mortality 25.3-fold [2]. In 2011, in Korea, Jung demonstrated a mortality rate 24 times higher in infants with congenital heart disease hospitalized for acute bronchiolitis compared to those without acute bronchiolitis [21]. In Canada, the Pediatric Investigator Collaborative Network on Infections in Canada demonstrated a mortality rate of 3.4% in children with pre-existing cardiac or pulmonary diseases [22]. Based on the results of these studies, the presence of acute bronchiolitis can lead to acute respiratory distress that is rapidly fatal in the absence of urgent and appropriate management.

In a study conducted by Che et al. in France on 29,784 infants under the age of one year hospitalized for acute bronchiolitis, of the eight infants who died, three had congenital heart disease, one had epilepsy, one had a chromosomal anomaly, and one had metabolic anomalies related to necrotizing enterocolitis [11]. In this study, the main cause of death due to viral bronchiolitis was decompensated congenital heart disease. Therefore, it seems important to carefully search for any associated comorbidity or underlying risk factor, particularly congenital heart disease or underlying pulmonary conditions, in any infants presenting with severe viral bronchiolitis to ensure appropriate prophylaxis.

We believe this study provides a clear picture of the current situation regarding viral bronchiolitis admissions in Morocco. At present, treatment for viral bronchiolitis remains focused on managing symptoms, primarily through measures such as nasal decongestion and spacing out meals to ease feeding difficulties. Given that RSV is the leading cause of viral bronchiolitis in infants, immunoprophylaxis stands out as the most effective way to reduce the significant burden of mortality, especially among high-risk infants, particularly those born prematurely or with congenital heart disease.

Among the pediatric population, there is no vaccine currently available for RSV. However, there are two immunoprophylaxis strategies, which are active immunoprophylaxis and monoclonal antibodies.

The active immunoprophylaxis involves administering a vaccine to pregnant women containing virus-like particles that include the RSV F protein [23]. This approach has been shown to reduce the risk of severe bronchiolitis by 70% during the first six months of life [24].

In cases of premature birth, infants cannot be protected by maternal immunization. Additionally, the seasonality of RSV may not align with the timing of maternal vaccination [25]. In view of these difficulties, the second strategy, based on passive immunoprophylaxis, remains highly relevant in preventing RSV-related viral bronchiolitis, particularly in high-risk groups. Currently, two monoclonal antibodies have demonstrated positive results. Palivizumab is a clinically approved monoclonal antibody that has been used for decades against RSV. The efficacy and safety of these have been demonstrated in three randomized clinical trials [24]. It is administered intramuscularly at a dose of 15 mg/kg once every 30 days, for a total of five doses.

In a meta-analysis involving 2831 high-risk newborns, specifically those born prematurely at less than 35 weeks of gestation, those with bronchopulmonary dysplasia, and those with congenital heart disease, palivizumab reduced RSV-related hospitalizations from 101 to 50 per 1000 population and decreased pediatric intensive care unit admissions from 34 to 17 per 1000 population [26]. In Korea, the use of palivizumab in children under one year of age with hemodynamically significant congenital heart disease has been implemented since 2009, reducing mortality rates to <1% [21]. Nirsevimab, another long-acting monoclonal antibody against RSV, is administered as a single dose and provides protection throughout the season. In a US study conducted in both the northern and southern hemispheres from November 2016 to November 2017 involving 1453 infants, Nivrsevimab was given to 969 infants, resulting in a 78.4% reduction in hospitalizations for LRIs linked to RSV [27].

In Morocco, palivizumab is the only prophylaxis available for use. Marketing authorization for palivizumab has been limited to three comorbidities: prematurity, bronchopulmonary dysplasia, and congenital heart disease. The need for monthly administration and high costs pose significant barriers to its universal use, particularly in developing countries such as Morocco.

Limitations

This is a retrospective study with all its difficulties. Medical records were not analyzed until after the patients had died. It would have been interesting to perform multiplex polymerase chain reaction (PCR) on infants in order to isolate the virus responsible and identify the viruses involved. The value of such isolation is mainly epidemiological and does not change the management of the disease.

## Conclusions

Viral bronchiolitis remains a major public health problem. It is responsible for high morbidity and mortality worldwide, but mainly in low-income countries. It is critical to look for comorbidities and/or underlying risk factors in all cases of severe viral bronchiolitis, particularly in premature babies and those with congenital heart disease. This approach can prevent serious or even fatal complications, lead to better-targeted preventive measures, and guarantee optimal referral and management. The exorbitant cost of RSV immunoprophylaxis is a major obstacle to its universal use, particularly in developing countries. The time has come to assess the value of using different preventive measures and to determine the most appropriate strategies for different target populations, namely, the value of monoclonal antibodies in preventing severe forms of viral bronchiolitis in infants with co-morbidities such as bronchopulmonary dysplasia and congenital heart disease.
